# Phosphoserine aminotransferase 1 is associated to poor outcome on tamoxifen therapy in recurrent breast cancer

**DOI:** 10.1038/s41598-017-02296-w

**Published:** 2017-05-18

**Authors:** Tommaso De Marchi, Mieke A. Timmermans, Anieta M. Sieuwerts, Marcel Smid, Maxime P. Look, Nicolai Grebenchtchikov, Fred C. G. J. Sweep, Jan G. Smits, Viktor Magdolen, Carolien H. M. van Deurzen, John A. Foekens, Arzu Umar, John W. Martens

**Affiliations:** 1 Department of Medical Oncology, Erasmus MC Cancer Institute, Erasmus University Medical Center, Rotterdam, The Netherlands; 2 Department of Laboratory Medicine, Radboud University Medical Center, Nijmegen, The Netherlands; 3 Department of Pathology, Admiraal de Ruyter Hospital, Goes, The Netherlands; 4 Department of Obstetrics and Gynecology, Technical University of Munich, Munich, Germany; 5 Department of Pathology, Erasmus University Medical Center, Rotterdam, The Netherlands; 6 Cancer Genomics Center Netherlands, Amsterdam, The Netherlands

## Abstract

In a previous study, we detected a significant association between phosphoserine aminotransferase 1 (PSAT1) hyper-methylation and mRNA levels to outcome to tamoxifen treatment in recurrent disease. We here aimed to study the association of PSAT1 protein levels to outcome upon tamoxifen treatment and to obtain more insight in its role in tamoxifen resistance. A cohort of ER positive, hormonal therapy naïve primary breast carcinomas was immunohistochemically (IHC) stained for PSAT1. Staining was analyzed for association with patient’s time to progression (TTP) and overall response on first-line tamoxifen for recurrent disease. PSAT1 mRNA levels were also assessed by reverse transcriptase quantitative polymerase chain reaction (RT-qPCR; n = 161) and Affymetrix GeneChip (n = 155). Association of PSAT1 to biological pathways on tamoxifen outcome were assessed by global test. PSAT1 protein and mRNA levels were significantly associated to poor outcome to tamoxifen treatment. When comparing PSAT1 protein and mRNA levels, IHC and RT-qPCR data showed a significant association. Global test results showed that cytokine and JAK-STAT signaling were associated to PSAT1 expression. We hereby report that PSAT1 protein and mRNA levels measured in ER positive primary tumors are associated with poor clinical outcome to tamoxifen.

## Introduction

Resistance to endocrine therapies is a major issue in recurrent estrogen receptor (ER) positive breast cancers^1^. Over the years several mechanisms have been connected to endocrine resistance, such as mutation in the ligand binding domain of the ER^2^, enhanced growth factor signaling, altered DNA methylation of cytosine phosphoguanine dinucleotides (CpG) of specific genes^3–7^, or the dysregulation of metabolic pathways^8, 9^. DNA methylation of CpG is an important mechanism to regulate gene expression in breast cancer^3, 4^, silencing tumor suppression genes (e.g. BRCA1)^5^ as well as genes involved epithelial-to-mesenchymal transition (EMT), and invasion^6, 7^. In a previous study, we have linked hyper-methylation in the promoter region of the PSAT1 gene, a key enzyme in serine synthesis, to a favorable outcome on tamoxifen treatment; conversely high PSAT1 mRNA levels were associated to tamoxifen resistance^10^. PSAT1 encodes an amino-transferase enzyme involved in the conversion of phospho-pyruvate, which is derived from oxidation of 3-phosphoglycerate, to phosphoserine. Phosphoserine is then converted into serine by the enzyme phosphoserine-phosphatase and further converted into glycine in order to feed the nucleotide biosynthesis pathway. Next to that the serine biosynthetic pathway itself has been shown to be a critical factor in breast cancer tumorigenesis^11^ and therapy resistance^10^. To further verify the predictive significance of PSAT1 as well as to translate the marker in into an assay which can easily be implemented in standard clinical practice we generated an immunohistochemical assay to quantitate PSAT1 protein levels in patient tissues and verified the association of the biomarker to tamoxifen therapy outcome. To this end, we assessed PSAT1 protein levels by IHC in a cohort of FFPE tissues and analyzed its association with tamoxifen therapy outcome. Furthermore, gene expression data of a cohort of ER positive breast carcinomas was used to gain insight into the role of PSAT1 in tamoxifen resistance.

## Results

**Figure 1:**
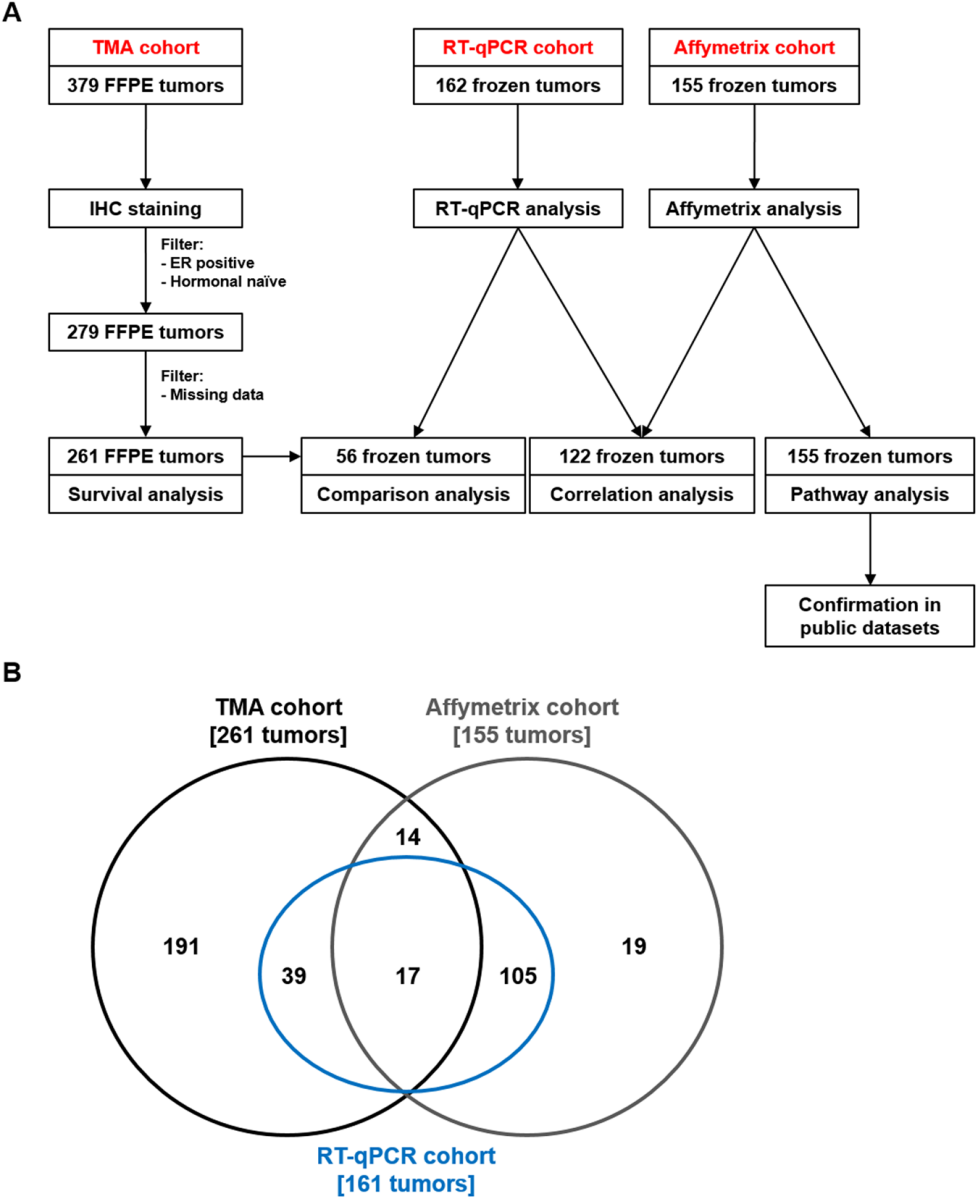
Schematic overview of experimental workflow. Panel A: a total of 379 FFPE tissues were captured on a tissue micro-array and analyzed by IHC. After filtering for ER positivity and hormonal naïve tumors, a total of 279 samples remained. Further filtering for missing data after IHC analysis yielded a panel of 261 tumors, on which survival analysis for the association of PSAT1 protein levels to TTP was performed. Parallel to this, PSAT1 mRNA expression was measured by RT-qPCR (n = 161) and Affymetrix chip (n = 155) approaches on frozen tumor specimens. These data were used for comparison between PSAT1 mRNA and protein levels (TMA and RT-qPCR; n = 56), correlation analysis (RT-qPCR and Affymetrix; n = 122), and pathway analysis (Affymetrix only; n = 155). Panel B shows tumor sample overlap between TMA, RT-qPCR and Affymetrix sets. Acronyms: ER: estrogen receptor; FFPE: formalin-fixed paraffin-embedded; IHC: immunohistochemistry; TMA: tissue microarray TTP: time to progression; RT-qPCR: quantitative reverse transcriptase polymerase chain reaction.

Schematic representation of analysis workflow is shown in Fig. 1.

### Association of PSAT1 protein to clinical variables

**Figure 2:**
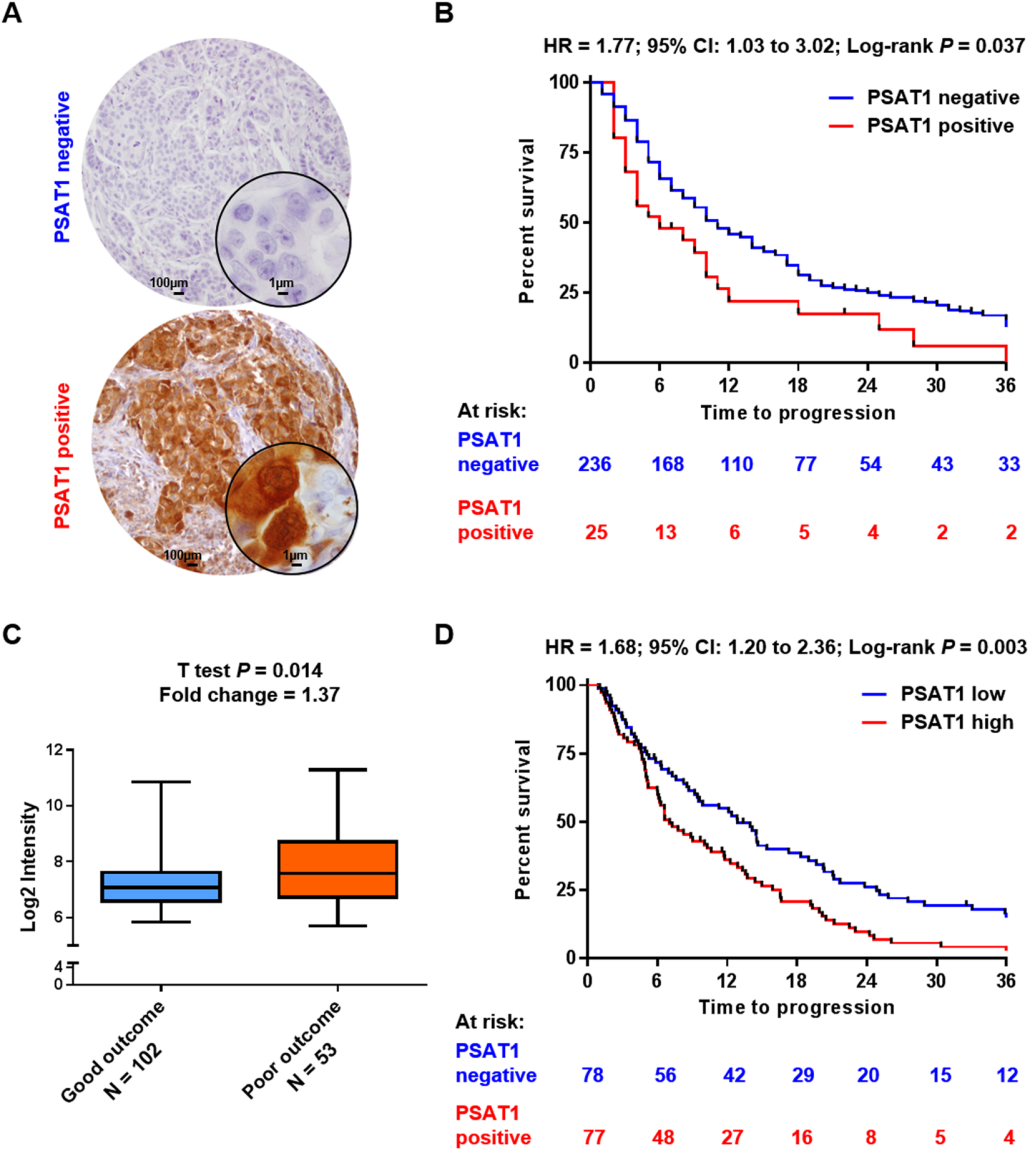
PSAT1 expression and clinical relevance in the TMA and Affymetrix datasets. Breast carcinoma IHC stained tissues either displayed high or low PSAT1 protein levels. Two representative specimen having either high or low PSAT1 are presented (panel A). Kaplan-Meier analysis showed that high expression of PSAT1 protein was significantly associated to shorter TTP when compared to tumors with low PSAT1 levels (panel B). PSAT1 mRNA levels were assessed by Affymetrix GeneChip. Statistical analysis not only showed PSAT1 mRNA expression was enrihed in poor outcome patients (t test *P* = 0.014; panel C), but Kaplan Meier analysis showed that, also in this set, PSAT1 expression was significantly associated to shorter TTP (panel D).

PSAT1 expression was observed in a small subset (*N* = 25) of ER positive breast tumors by IHC, while the majority of cores did not show any PSAT1 expression (*N* = 236). Examples of PSAT1 protein IHC stainings are shown in Fig. 2A. PSAT1 was expressed both in the cytoplasms as well as in the nucleus of carcinoma cells, with no difference between the two stainings in terms of intensity and quantity of stained cells (data not shown). Testing for association of PSAT1 protein expression to clinical and histo-pathological parameters showed that PSAT1 expression was significantly associated with poor grade tumors (χ^2^ test for trend *P* < 0.001) and local relapse (χ^2^ test *P* = 0.009; Table 1). Taken together, these data suggest that PSAT1 expression is higher in tumors with poor differentiation that tend to relapse locally.

**Table 1:** Association of PSAT1 protein expression to clinical and histo-pathological characteristics.

	Patients included in analysis	PSAT1 negative	PSAT1 positive	*P* ^‡^
Total	261 (100.0)	236 (100.0)	25 (100.0)	
Age^*^				
≤55 years	99 (37.9)	89 (37.7)	10 (40.0)	0.831
>55 years	162 (62.1)	147 (62.3)	15 (60.0)	
Menopausal status^*^				
Premenopausal	68 (26.1)	61 (25.8)	7 (28.0)	0.813
Postmenopausal	193 (73.9)	175 (74.2)	18 (72.0)	
Tumor size				
T1 (≤2 cm)	112 (42.9)	101 (42.8)	11 (44.0)	0.899
T2 (2–5 cm) + Tx	127 (48.7)	115 (48.7)	12 (48.0)	
T3 (>5 cm) + T4	22 (8.4)	20 (8.5)	2 (8.0)	
Tumor differentiation^**,†^				
Good	42 (16.1)	41 (75.0)	1 (4.0)	<0.001
Moderate	143 (54.8)	136 (57.6)	7 (28.0)	
Poor	75 (28.7)	58 (24.6)	17 (68.0)	
Involved lymph nodes^†^				
0	87 (33.3)	78 (33.1)	9 (36.0)	0.822
≥1	166 (63.6)	151 (64.0)	15 (60.0)	
Disease free interval				
≤12 months	45 (17.2)	40 (16.9)	5 (20.0)	0.780
>12 months	216 (82.8)	196 (83.1)	20 (80.0)	
Dominant site of relapse				
Loco-regional	28 (10.7)	22 (9.3)	6 (24.0)	0.009
Bone	104 (39.8)	101 (42.8)	3 (12.0)	
Visceral	56 (21.5)	50 (21.2)	6 (24.0)	
Bone and other	73 (28.0)	63 (26.7)	10 (40.0)	
PgR^†^				
Negative	69 (26.4)	63 (26.7)	6 (24.0)	1.000
Positive	191 (73.2)	172 (72.9)	19 (76.0)	

*Age and menopausal status were assessed at start of tamoxifen therapy.

**Tumor differentiation was evaluated through Scarff-Bloom-Richardson grading system.

^†^Missing data not reported.

^‡^PSAT1 association to clinical parameters was assessed by Fisher’s exact test (age, menopausal status, number of involved lymph nodes, disease free interval and PgR), χ^2^ test (dominant site of relapse), and χ^2^ test for trend (tumor size, grade).

Acronyms: PgR: progesterone receptor.

Subsequently, in order to evaluate whether PSAT1 IHC stainings (i.e. negative vs positive) were associated to patient outcome, Cox and logistic regression analyses were performed on the 261 stained tissue cores. PSAT1 expression was significantly associated to shorter TTP both in univariate (HR = 1.77; 95% CI: 1.77 to 3.03; *P* = 0.037; Fig. 2B) and multivariate analyses (HR = 1.63; 95% CI: 1.02 to 1.59; *P* = 0.039; Table 2) independent of traditional predictive factors (DFI, dominant site of relapse and tumor differentiation). A trend was observed in univariate logistic regression analysis for the association of PSAT1 protein levels to clinical benefit (i.e. CR + PR + SD; OR = 0.45; 95% CI: 0.19 to 1.02; univariate Logistic regression *P* = 0.057 Table S1), while no association to objective response (i.e. CR + PR only) was observed (OR = 0.26; 95% CI 0.15 to 1.81; univariate Logistic regression *P* = 0.304; Table S2). The lack of association to clinical response criteria is maybe due to the small amount of tumors displaying PSAT1 expression. Overall, these data provide evidence that PSAT1 protein expression is associated to short time to progression in patients treated with tamoxifen for recurrent disease.

**Table 2:** Cox regression analysis for TTP of PSAT1 stained tumors.

	n of patients	Univariate			Multivariate		
		HR	95% CI	*P*	HR	95% CI	*P*
**PSAT1**							
Negative	236	1.00			1.00		
Positive	25	1.77	1.03 to 3.03	0.037	1.63	1.02 to 2.59	0.039
**Age** ^*^							
≤55 years	99	1.00			1.00		
>55 years	162	0.50	0.38 to 0.65	<0.001	0.56	0.42 to 0.73	<0.001
**Disease free interval**							
≤12 months	41	1.00					
>12 months	220	0.69	0.49 to 0.98	0.040	0.60	0.42 to 0.86	0.006
**Dominant site of relapse**							
Loco-regional	28	1.00					
Bone	104	1.68	1.05 to 2.68	0.030	1.91	1.17 to 3.09	0.009
Visceral	56	1.53	0.92 to 2.53	0.100	1.87	1.11 to 3.13	0.017
Bone and other	73	1.80	1.10 to 2.93	0.019	1.93	1.17 to 3.17	0.009
PgR^**^							
Negative	69	1.00					
Positive	191	0.80	0.60 to 1.07	0.134			
**Her2 status** ^**^							
Negative	210	1.00					
Positive	49	1.20	0.87 to 1.67	0.265			
**Tumor differentiation** ^**^							
Good	42	1.00			1.00		
Moderate	143	1.65	1.13 to 2.41	0.010	1.50	1.02 to 2.22	0.040
Poor	75	2.42	1.60 to 3.67	<0.001	2.03	1.30 to 3.16	0.002

^*^Age was assessed at start of tamoxifen therapy.

^**^Missing data not reported.

### Comparison between PSAT1 mRNA and protein levels

In order to assess whether PSAT1 mRNA and protein expression were associated, tumors in which PSAT1 mRNA was measured by RT-qPCR (n of overlap = 56) were stratified according to IHC results (i.e. PSAT1 positive and PSAT1 negative). A significant difference (Mann-Whitney *P* = 0.009) was observed between mRNA levels of PSAT1 positive and negative tumors (Fig. S1A). Comparison between mRNA levels measured by Affymetrix platform and stratified according to IHC data (i.e. absent vs. present) did not show any significance (Mann-Whitney *P* = 0.133), probably due to imbalances in group distributions (i.e. PSAT1 positive = 3; PSAT1 negative = 28; Fig. S1B) and the small overlap between Affymetrix and TMA sets. PSAT1 mRNA levels measured by RT-qPCR and Affymetrix in 122 tumors showed moderate-strong correlation (Spearman r = 0.74; *P* < 0.001; Fig. S2). These data confirm that PSAT1 levels (mRNA and protein) show a similar trend when measured by either RT-qPCR or IHC.

### Clinical significance of PSAT1 in the gene expression cohort and pathway analysis

**Figure 3:**
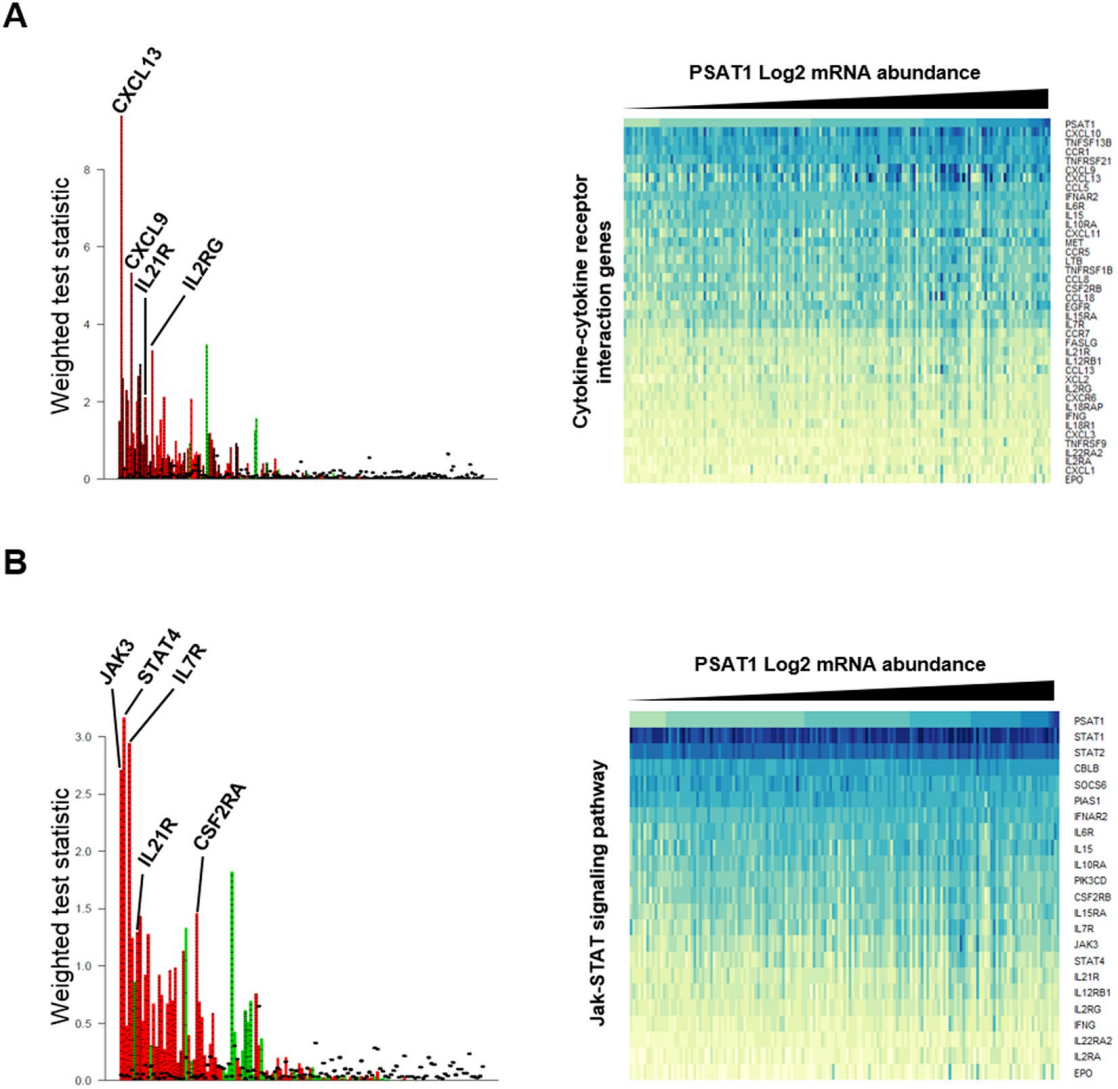
PSAT1 expression associated genes in the gene expression dataset. The 155 tumors in the Affymetrix cohort were stratified according to PSAT1 expression. All genes were annotated for KEGG terms and Global test was performed to assess which terms were associated to PSAT1 expression. Panel A and B display the top 2 KEGG pathways associated to PSAT1: Cytokine-cytokine receptor interaction (**A**) and Jak-STAT signaling pathway (**B**). Bar charts (left) represent enriched genes in each pathway, with red and green columns representing the association to high and low expression of PSAT1, respectively. Heatmaps of most significantly enriched genes in each pathway (enrichment statistic *P* < 0.01; genes are ordered based on decreasing average expression) in relation to PSAT1 expression are also shown (right).

We used an Affymetrix GeneChip analyzed dataset (n = 155; 102 × good outcome; 53 × poor outcome) in order to define which genes were associated to PSAT1. First, we evaluated the expression of key breast cancer markers: ESR1 gene was found significantly downregulated in the poor outcome group after tamoxifen therapy (t test *P* = 0.009; fold change = 0.67). Key breast cancer prognostic markers, such as PGR (t test *P* = 0.168; fold change = 0.76) and ERBB2 (t test *P* = 0.123; fold change = 1.23), showed no change between the two patient categories. When assessing PSAT1 levels, a significant enrichment was found in the poor outcome patient group (t test *P* = 0.014; fold change = 1.37; Fig. 2C). In order to confirm that PSAT1 expression was associated to shorter TTP, we stratified patients in PSAT1 low and high expressing groups (cutoff: median expression; median Log2 intensity = 7.086) and performed survival analysis. Kaplan-Meier curves showed that patients whose tumors expressed high levels of PSAT1 suffered from faster tumor progression when compared to the PSAT1 low group (HR = 1.68; 95% CI: 1.20 to 2.36; Log-rank *P* = 0.003; Fig. 2D). The set was stratified based on median PSAT1 levels (categories: PSAT1 low, PSAT1 high), and then analyzed by global test in order to investigate which (KEGG) pathways were associated to PSAT1 expression. A total of 8 pathways were significantly associated to PSAT1 expression (adjusted Holm-Bonferroni *P* < 0.05; Table S3). As expected, the serine, glycine and threonine metabolism (*P* < 0.001; Table S4) was the most enriched pathway which, in addition to PSAT1, showed enrichment of its upstream enzyme PHGDH. In addition to this, two pathways stood out from the rest, which comprised a large number of genes associated to high PSAT1 expression: cytokine-cytokine receptor interaction (*P* = 0.001; Fig. 3A; Table S5) and JAK-STAT signaling (*P* = 0.004; Fig. 3B; Table S6) pathways. The cytokine-cytokine receptor interaction pathway comprised molecules involved in mesenchymal cell growth and immune cell signaling, such as PDGFRA and IL4R, respectively. On the other hand, most of the genes associated to the JAK-STAT signaling pathway comprised molecules involved in immune cell signaling, such as JAK3 and IL21R. To confirm these results, we analyzed a second independent gene expression dataset derived from the combination of three public sets (GSE2990, GSE7390 and GSE11121). For this dataset ER positive and lymph node negative tumors were selected (n = 404) and stratified according to PSAT1 expression and subgroups (i.e. PSAT1 low vs PSAT1 high) were analyzed by global test against the KEGG database. The Cytokine-Cytokine receptor interaction (Holm-Bonferroni *P* = 1.45E-04; Table S7 and Fig. S3A) and Jak-STAT signaling (Holm-Bonferroni *P* = 1.75E-07; Table S8 and Fig. S3B) pathways were found significantly enriched also in this set, thus corroborating our previous analysis. These data provide a link between the involvement of immune cell signaling and the expression of the Serine metabolism pathway enzyme PSAT1.

To further assess the association of PSAT1 to presence of immune cells we derived the expression levels of a 152-gene signature which we previously associated to tumor infiltrating lymphocytes (TILs) in breast cancer^12, 13^. We calculated the average TIL-score (see Material and Methods section) and evaluated its correlation to PSAT1 mRNA expression in our 155 samples gene expression dataset. A significant, albeit weak, correlation between the TIL signature and PSAT1 expression was found (Spearman r = 0.239; *P* = 0.003; Fig. S4A). Furthermore, PSAT1 was found to be significantly enriched in the high TIL signature expression group (median cutoff; Mann Whitney *P* = 0.009; Fig. S4B). Taken together, these data indicate that PSAT1 might be related not only to differential cell metabolism reprogramming, but also to immune cell signaling and lymphocyte tumor infiltration.

## Discussion and Conclusions

Resistance to tamoxifen therapy is a major cause of death in ER positive recurrent breast cancer^14^. Several molecular mechanisms have been associated to tamoxifen resistance, such as point mutation in the ER, dysregulation of ER-mediated gene expression, or cross-talk mechanisms involving growth factor and PI3 kinase signaling^2, 15, 16^. However, few of these have been verified on clinical specimens. In a previous study, PSAT1 inactivation by promoter methylation, and consequently low mRNA levels, have been associated to good outcome to tamoxifen treatment^10^. We have here generated an IHC assay to measure PSAT1 protein levels in breast cancer tissues and evaluated whether PSAT1 protein levels reflected previous findings and mRNA analysis in the association to tamoxifen therapy outcome. In addition to this, we used gene expression data from a large cohort of ER positive breast cancer patients to further confirm PSAT1 association to short TTP at the mRNA level, compare PSAT1 protein and mRNA levels, and performed global test analysis to potentially elucidate molecular mechanisms related to PSAT1 expression and tamoxifen resistance.

Our IHC staining of a large cohort of paraffin-embedded breast cancers showed that only a small group of specimens displayed detectable PSAT1 protein expression. Statistical analysis of IHC stainings showed that PSAT1 expression was associated to poor tumor differentiation and loco-regional metastases. Furthermore, Cox regression analyses showed that PSAT1 expression was significantly associated to shorter TTP independently of other predictive factors (e.g. DFI). On the other side, logistic regression analyses showed that PSAT1 expression showed no association to clinical benefit or objective response, probably due to the fact that only a small portion of tumors displayed detectable PSAT1 protein expression. Small subset size could have also influenced the comparison between PSAT1 protein and mRNA levels measured by Affymetrix GeneChip technology, leading to inconclusive results. Despite of this, comparison between PSAT1 IHC and RT-qPCR expression showed a significant association. This is probably due to the fact that, despite the small amount of samples overlapping between the TMA and the RT-qPCR sets, more accurate mRNA quantitation can be achieved through RT-qPCR compared to microarray technology.

Having established that PSAT1 protein levels are indicative of fast tumor progression tumors treated with first line tamoxifen, we sought to investigate which molecular pathways were associated to PSAT1 expression in our Affymetrix dataset. In this set, PSAT1 was found enriched in poor outcome patients and its expression was also significantly associated to shorter TTP. Global test analysis showed that, apart from the glycine, serine and threonine metabolism pathway, the JAK-STAT signaling and the cytokine-cytokine receptor interaction pathways were positively associated to PSAT1 expression. Molecules of interest in the cytokine pathway comprised IL4R and IL21R, which were enriched in poor outcome patients and positively associated to PSAT1 expression. IL4R has been associated to metabolic reprogramming of tumor cells in favor of an increased glucose uptake and enhancement of glutamine metabolism^17^, while IL21R expression has been previously linked to breast cancer cell proliferation and migration in MM231 breast cancer cell lines^18^. Furthermore, IL21 binding to its receptor promotes the activation of several signaling cascades, such as the JAK-STAT pathway, with differential effects related to tissue of expression^19, 20^. As both the JAK-STAT and cytokine pathways are directly linked to inflammation and contribute to tumor growth and spread in several cancer types^21, 22^, we aimed at investigating the relation between infiltrating tumor cells and PSAT1 expression. Our analyses resulted in a significant association of PSAT1 levels to a set of genes predictive of TIL infiltration in breast cancer^12, 13^, indicating a possible involvement of immune cells in PSAT1 expression and tamoxifen therapy resistance.

Glucose and glutamine are the main molecules that cells metabolize to generate ATP through glycolysis and the tricarboxylic acid cycle (TCA). During anabolic states cancer cells reprogram their metabolic pathways, from oxidative phosphorylation to glycolysis and serine production^23, 24^. Out of the latter, serine can be further converted into Glycine and subsequently in purines, while intermediate products such as α-ketoglutarate can be directed towards the TCA cycle^25^. PSAT1 has a key role in serine biosynthesis, by catalyzing the oxidation of 3-phosphohydroxypyruvate into phosphoserine using glutamate. Previous studies have shown that the Serine pathway has a key role in cancer metabolism, using glycolysis-derived glucose for serine production and tumor growth^24^. PSAT1 and its related molecules (e.g. its upstream enzyme PHGDH) have been shown to be overexpressed in ER negative tumors and cell lines, conferring a metabolic-related growth advantage through production of alpha-ketoglutarate and altering the cell redox status by redirecting 3-phosphoglycerate^11, 26^. In the perspective resistance to tamoxifen, and following our global test results, the metabolic switch of tumor cells toward serine production may be an indication of a vaster tumor cell and tumor microenvironment reprogramming, involving cytokine and JAK-STAT signaling. As JAK-STAT signaling has been shown not only to induce metabolic switches in tumor cells, but also facilitates cell cycle progression by promoting the activation of cyclin dependent kinases^22, 27, 28^, it is possible that PSAT1 enrichment is indicative of such upstream signaling. Still, functional studies should be performed in order to establish the hierarchical link between metabolic reprogramming, JAK-STAT signaling, and resistance to tamoxifen therapy.

We here conclude that PSAT1 protein expression is associated to rapid progression of metastatic breast cancer treated with first line tamoxifen and that it is likely associated to general tumor cell metabolic reprogramming, probably through IL-JAK-STAT signaling. Following validation in prospective cohorts, evaluation of PSAT1 could potentially become a clinically actionable biomarker. On the other hand, functional experiments (e.g. co-culture of tumor and lymphocytic cells in presence of anti-estrogens) could further elucidate the role of this protein in tamoxifen resistance.

## Materials and Methods

### Patient cohorts

This study used previously described FFPE tumor tissues which were incorporated into a TMA (n = 379)^29^. Breast cancer tissues derived from patients who underwent tumor resection (between 1985 and 2000) and were treated with first line tamoxifen for recurrent disease, were included in the study. A specialized breast pathologist (CHMvD) reviewed all the primary tumor tissue histologic subtypes according to the world health organization (WHO) and histologic grade was defined according to the modified Bloom-Richardson score, which takes into account tubule formation, mitotic activity and nuclear pleiomorphism^30^. Out of this set, only ER positive primary tumors from patients who did not receive any adjuvant (i.e. post-surgical resection) hormonal therapy were included for statistical analysis. This led to the inclusion of 279 tissues derived from patients with ER positive primary tumors (Table S9), of whom response data were collected according to the standard International Union Against Cancer criteria^31^. A total of 10 (3.58%) and 42 (15.05%) patients showed complete (CR) and partial remission (PR), respectively. One hundred and fifty-seven patients showed no change (NC) of disease, of whom 27 (9.68%) displayed NC for less (≤) than 6 months, while 130 (46.59%) showed NC for longer (>) than 6 months (defined as stable disease [SD]). A total of 70 (25.09%) patients displayed progressive disease (PD). Clinical benefit was defined as CR + PR + SD patients (n = 182; 65.22%), while objective response was defined as CR + PR only (n = 52; 18.63%).

For gene expression analysis a total of 155 ER positive fresh frozen primary breast carcinomas were collected, which derived from patients treated with tamoxifen therapy upon disease recurrence. Total RNA was analyzed by GeneChip® Human Genome U133 Plus 2.0 and Perfect Match Arrays (Affymetrix, Santa Clara, CA, USA). Dataset is available from gene expression omnibus database (ID: GSE82173). As previously performed^32, 33^, tamoxifen therapy outcome groups in this set were defined based on TTP: patients displaying progression before (≤) 6 months after start of tamoxifen therapy were classified as poor outcome, while patients that showed progression of disease after (>) 6 months were defined as good outcome. This set comprised 102 good and 53 poor outcome patients, respectively (Table S10). For a subset of these tumors (n = 122), and for 39 additional specimens only included in the TMA, quantitative reverse transcriptase PCR (RT-qPCR) data obtained from total RNA isolated from ER positive fresh frozen primary breast carcinomas was also available (Fig. 1B).

This study used coded primary tissues, in accordance with the Code of Conduct of the Federation of Medical Scientific Societies in the Netherlands (http://www.federa.org/codes-conduct). Reporting Recommendations for Tumor Marker Prognostic Studies were followed^34^. Informed consent was obtained for the use of clinical specimens. Ethical approval of this study was obtained from the Medical Ethics Committee of the Erasmus Medical Center, The Netherlands (MEC 02.953).

### Tissue micro-array

TMA was prepared using an ATA 27 (Beecher Instruments, Sun Prairie, WI, USA). 379 FFPE ER positive breast cancer tissues derived from patients who received tamoxifen as first-line therapy for recurrent disease were used to prepare the TMA. For each paraffin block, tumor area was delineated by a specialized breast pathologist (CHMvD), from which tissue cores of 0.6 mm were collected and transferred in triplicate in a recipient TMA block. For each tumor, three different punches were taken within the marked tumor area. TMA slides were digitalized and analyzed using Slidepath software (Leica Microsystems, Solms, Germany).

### Anti-PSAT1 antibody

In order to select protein surface exposed epitopes for antibody production, prediction models were generated in expasy (http://expasy.org/) and matched with publicly available crystal structures of *E. coli* and *B. alcalophilus* PSAT1 homologues. Putative epitope regions were selected based on absence of secondary structures (i.e. excluding regions involved in α-helix or β-strand structures) and being present in both PSAT1 splice variants^35^. Two peptides were selected in total: PSAT1-A (DYKGVGISVLEMSHRSS, aa 31–47) and PSAT1-B (KLGSYTKIPDPSTWNLNP; aa 127–144). Both peptides were synthetized adding a Cys residue at the N-terminus for disulfide linkage to keyhole limpet hemocyanin (KLH). Rabbit pre-immunization sera were tested for absence of immune reaction by Western Blot analysis against full length recombinant PSAT1 (sequence including exon 8). KLH-conjugated peptides were injected into rabbits, which received boost immunizations at day 20, 30, 40, 61, 75, 90, and 104. Sera were then collected at day 120. Peptide synthesis, conjugation to KLH, and rabbit immunization steps were performed by Pineda Antibody Service (Berlin). For antibody purification, immunoaffinity columns were prepared using recombinant human full length PSAT1 (including exon 8). Recombinant PSAT1 was expressed in *E. coli* as a N-terminal histidine tagged protein and was purified via Ni^2+^-NTA (Qiagen) affinity chromatography under denaturing and slightly reduced conditions (purity > 95%). After protein refolding, PSAT1 was recovered in soluble form in a 1x PBS and 1 mM DTT solution.

PSAT1-linked column was prepared by mixing ~2 mL of AffiGel-10® and AffiGel-15 (BioRad) in a 1:1 ratio. Sorbent mixture was then pre-treated by sequential washings: 10 mL of isopropanol, 5 mL deionized water, and 5 mL of 50 mM HEPES buffer in deionized water (pH 7.4). A total of 0.5 mg of soluble recombinant PSAT1 dissolved in 5 mL of 50 mM HEPES was mixed with the sorbent and incubated overnight at 4 °C. Sorbent was then washed with 1x PBS (5 × 5 mL), followed by blocking solution incubation (100 mM glycine in 1x PBS) for 1 h at room temperature. Further washings with 1 mL of 4 M guanidinium chloride in 1x PBS (1 × 5 mL) and 40 mL of 1x PBS (1 × 5 mL) were performed. Rabbit-derived blood was centrifuged at 1711 g for 10 min and 4 mL of rabbit serum were injected onto the sorbent (three sequential times), followed by a 25 mL 1x PBS wash. Antibodies were then eluted with 4 mL of 20 mM glycine in 1x PBS (pH 2.4), and collected in tubes to which pH compensatory solution (200 µL of 1x PBS; pH 11.5) was added in order to achieve pH 7.5. Protein concertation was assessed by measuring absorbance at 260/280 nm. Eluate aliquots were then mixed with 100% v/v glycerol in a 1:1 ratio and stored at −20 °C. Peptide synthesis, conjugation to KLH, and rabbit immunization steps were performed by Pineda Antibody Service (Berlin; permission: Veterinary Inspection Office; County: Barmin, Brandenburg, Germany; file reference. 39TSch41/14).

### Immunohistochemistry

FFPE tissue sections (5 µm) captured on the TMA were incubated at 60 °C (30 min) and washed in xylene (3 × 5 min) for de-paraffination. Decreasing concentrations of ethanol were used for tissue re-hydration: 100% ethanol (1 × 5 min, 2 × 2 min), 70% ethanol (1 × 2 min), 50% ethanol (1 × 2 min), distilled water (1 × 2 min). Incubation with DAKO (Agilent Technologies Inc, Glostrup, Denmark) antigen retrieval (pH 6.0) solution diluted 1:10 in MilliQ water was performed at 95 °C for 40 min. After cooling down to room temperature, slides were subjected to sequential washes: 1x PBS (3 × 5 min), 0.003% H_2_O_2_ in 1x PBS (i.e. blocking of endogenous peroxidase activity; 1 × 10 min), 1x PBS (3 × 5 min), 5% BSA in 1x PBS (i.e. blocking solution; 1 × 30 min). Anti-PSAT1 rabbit polyclonal primary antibody diluted 1:40 in Dako antibody diluent was added to slides and incubated overnight at 4 °C. Slides were then washed with 1x PBS (3 × 5 min), and DAKO Envision® labelled polymer HRP-Rabbit was added to each slide (200 µl per slide) and incubated for 45 min at room temperature. Sequential washes were performed as follows: 1x PBS (3 × 5 min), 1:15 solution of DAB + chromogen in DAB + substrate buffer (100 µl per slide; 1 × 10 min in the dark). Further hematoxylin/eosin stainings were performed as follows: tap water (1 × 5 min), hematoxylin (1 × 1 min), eosin (1 × 1 min), 50% ethanol (2 × 2 min), 70% ethanol (2 × 2 min), 100% ethanol (2 × 2 min, 1 × 5 min), and xylene (2 × 2 min, 1 × 5 min). Cover glasses were mounted on slides with Pertex and were left to dry. Slides were digitalized and analyzed using Slidepath software (Leica Microsystems, Solms, Germany). PSAT1 stained breast carcinoma cells were scored by an experienced researcher in a blind manner for both intensity (i.e. negative; weak; weak-moderate; moderate; moderate-strong; strong) and quantity (i.e. 0%; 1–5%; 6–10%; 11–25%; 26–50%; >50%) of stained carcinoma cells. Triplicate scores were then verified and consolidated by a second experienced researcher, which was extensively trained by a specialized breast pathologist.

Breast cancer cell lines were incorporated in the TMA as control specimens. PSAT1 protein expression measured by IHC was compared to mRNA levels measured by RT-qPCR (for method see below). Comparison between PSAT1 protein and mRNA levels in breast cancer cell lines is displayed in Fig. S4.

### RNA extraction and RT-qPCR analysis

To measure mRNA transcript levels, total RNA was isolated using RNA-Bee (Campro Scientific, Veenendaal, The Netherlands) according to the manufacturer’s instructions, as previously described^36^. For RT-qPCR, complementary DNA synthesis (RT reaction) was generated using the RevertAid H Minus First Strand cDNA Synthesis Kit (Thermo Scientific, Amsterdam, The Netherlands), followed by incubation at 37 °C with 0.1 U/µL ribonuclease H (Thermo Scientific) for 30 min. RT-qPCRs were performed on cDNA generated from 10 ng total RNA and normalized using the dCq method on the average of 3 reference genes (*HMBS*, *HPRT1* and *TBP*). Quantification of PSAT1 was performed using the TaqMan probe–based gene expression assay Hs00253548_m1 specific for splice variant beta (Applied Biosystems/Life Technologies, Warrington, WA, USA) as previously described^10, 36^, For Affymetrix gene expression profiling, total RNA samples were cleaned and DNAse treated with the NucleoSpin RNA II kit according the manufacturers instruction (Machery-Nagel, Dueren, Germany) and shipped to ServiceXS (Leiden, The Netherlands) for downstream processing with the 3’IVT express kit and hybridization on the Human Genome (HG) U133 Plus 2.0 array (n = 20) and HG U133 Perfect Match.

### Statistical analysis of IHC data

PSAT1 TMA scores were stringently filtered for missing data points (i.e. lack of triplicate analysis per tissues, which derived from either lack of core or lack of enough [>30] tumor cells in at least one core), which led to a set of 261 tissue samples. Due to the fact that PSAT1 positive cases showed strong and ubiquitous staining, PSAT1 protein expression was scored as either absent (i.e. PSAT1 negative: 0% stained carcinoma cells) or present (i.e. PSAT1 positive: ≥1% stained carcinoma cells). PSAT1 (negative/positive) association to clinical and histo-pathological characteristics was assessed by Fisher’s exact test (i.e. age, menopausal status, number of positive lymph nodes, disease free interval [DFI], and progesterone receptor status), χ^2^ test (dominant site of relapse), and χ^2^ test for trend (tumor size, tumor differentiation). Statistical tests p values are displayed in Table 1. Association of PSAT1 expression to TTP and response criteria was tested by Cox and logistic regression, respectively. Patient age (cutoff: 55 years), disease free interval (cutoff: 12 months), dominant site of relapse (bone, visceral, loco-regional, bone and other), progesterone receptor (PgR) positivity, Her2 overexpression, and degree of tumor differentiation (Bloom-Richardson; good, moderate, poor) were included in all regression analyses. Variables that did not display any significant association with TTP or any response criteria (i.e. *P* ≥ 0.05) were excluded from the multivariate regression model. Cox regression, logistic regression, HRs, odds ratios (OR), and 95% CIs were calculated in Stata (v 13.1; Stata Corp, College Station, TX, USA).

### Gene expression analysis

Raw.CEL files for both Affymetrix platforms were processed using fRMA with ‘robust weighted average’ (i.e. HG U133 Plus 2.0; n = 20) and ‘random effect’ (i.e. HG U133 Perfect Match; n = 135) as summarization methods. fRMAvecs from the Plus 2.0 array were used for both batches^37^. Due to the fact that this set was measured in 2 separate batches, ComBat algorithm-based normalization was applied [26]. The 155 samples gene identifiers and Affymetrix probe intensities were imported in Microsoft Excel. For probes annotated to the same genes, probes were selected based on highest variability and median level, leading to a final list of 19,042 reliable probes.

### Statistical and pathway analyses of gene expression data

To assess whether PSAT1 protein and mRNA levels measurements were comparable, PSAT1 Log2 probe intensities and RT-qPCT C_q_ values were classified according to the IHC score (categories: negative vs. positive), and tested for differences by Mann-Whitney test. To assess whether PSAT1 mRNA levels measured by Affymetrix and RT-qPCR were comparable, Spearman correlation analyses was performed.

In order to assess whether pathway analysis would achieve not only biological, but also clinical significance, patients were stratified according to median PSAT1 expression (i.e. low: Log2 intensity < median; high: Log2 intensity ≥ median). Survival curves were plotted and differences in TTP were assessed by Logrank test. Association of KEGG^38^ pathways to PSAT1 and its associated genes was performed through global test^39, 40^. Significant pathways out of global test were selected based on Holm-Bonferroni corrected significance (*P* < 0.05). Most enriched genes in each pathway (enrichment statistic *P* < 0.01) were selected and their abundances plotted in relation to PSAT1 mRNA levels (genes were ordered based on average intensity across samples).

In order to verify our global test results in an independent cohort, public datasets containing Affimetrix microarray (v133a) data of breast cancer patient derived tissues (GSE2990, n = 189; GSE7390, n = 198; and GSE11121, n = 200) were downloaded for downstream analysis. The three datasets were combined, probes were summarized using fRMA, as previously performed for our in-house dataset, and batch effects were corrected using ComBat. Subsequently, patients were filtered for ER status and nodal status (i.e. only ER positive, lymph node negative cases were selected, resulting in a total of 404 cases) and stratified according to median PSAT1 expression in PSAT1 high and PSAT1 low groups. Global test analysis of median PSAT1 stratified samples (against KEGG pathways) was then performed.

PSAT1 mRNA expression was also associated to a 152-gene signature associated to tumor infiltrating lymphocytes (TIL)^12, 13^. Average expression of the TIL gene signature was calculated for each sample to derive a global TIL-score for every sample. PSAT1 association to the TIL signature was evaluated by Spearman correlation. Furthermore, difference in PSAT1 mRNA expression between high and low TIL-score (median cutoff) was evaluated by Mann-Whitney test.

Log-rank, Mann-Whitney and Spearman correlation analyses were all performed in GraphPad (v5.0). Global test bar charts and heatmaps were generated in R (v3.3.2).

## Electronic supplementary material

Supplementary material

Table S4

Table S5

Table S6

Table S7

Table S8
