# Supplementary material for: Phosphoserine aminotransferase 1 is associated to poor outcome on tamoxifen therapy in recurrent breast cancer
Source: Sci Rep. 2017 May 18;7:2099. doi: 10.1038/s41598-017-02296-w (PMC5437008; doi:10.1038/s41598-017-02296-w)

Title: “Phosphoserine Aminotransferase 1 is associated to poor outcome on tamoxifen therapy in recurrent breast cancer”

Tommaso De Marchi[a,*,†], Mieke A. Timmermans[a,*], Anieta M. Sieuwerts[a,g], Marcel Smid[a], Maxime P. Look[a], Nicolai Grebenchtchikov[b], Fred C.G.J. Sweep[b], Jan G. Smits[c], Viktor Magdolen[d], Carolien H.M. van Deurzen[e], John A. Foekens[a], Arzu Umar[a], and John W. Martens[a,f,†].

Affiliations:

1. Department of Medical Oncology, Erasmus MC Cancer Institute, Erasmus University Medical Center, Rotterdam, The Netherlands
2. Department of Laboratory Medicine, Radboud University Medical Center
3. Department of Pathology, Admiraal de Ruyter Hospital, The Netherlands
4. Department of Obstetrics and Gynecology, Technical University of Munich, Germany.
5. Department of Pathology, Erasmus University Medical Center, Rotterdam, The Netherlands
6. Cancer Genomics Center Netherlands, Amsterdam, The Netherlands

* These authors contributed equally to the study.

† These are the corresponding authors.

Table of contents:

a. Supplementary tables (Page S-2)

b. Supplementary Figure Legends (Page S-7)

c. Supplementary Figures (Page S-9)

Table S-1. Logistic regression analysis for clinical benefit of PSAT1 stained tumors.

		Univariate			Multivariate		
	N	**OR**	**95% CI**	***P***	**OR**	**95% CI**	***P***
**PSAT1**							
**Negative**	236	1.00					
**Positive**	25	0.45	0.19 to 1.02	0.057			
**Age***							
**≤ 55 years**	99	1.00			1.00		
**> 55 years**	162	2.02	1.19 to 3.40	0.009	1.74	1.01 to 2.99	0.046
**Disease-free survival**							
**≤ 12 months**	41	1.00					
**> 12 months**	220	1.43	0.72 to 2.82	0.307			
**Dominant site of relapse**							
**Loco-regional**	28	1.00					
**Bone**	104	0.75	0.30 to 1.88	0.548			
**Visceral**	56	0.67	0.25 to 1.78	0.419			
**Bone and other**	73	0.77	0.30 to 1.99	0.587			
**PgR****							
**Negative**	69	1.00					
**Positive**	191	1.31	0.74 to 2.31	0.358			
**Her2 status****							
**Negative**	210	1.00			1.00		
**Positive**	49	0.51	0.27 to 0.95	0.035	0.65	0.33 to 1.26	0.199
**Tumor differentiation****							
**Good**	42	1.00			1.00		
**Moderate**	143	0.46	0.20 to 1.08	0.076	0.52	0.22 to 1.22	0.131
**Poor**	75	0.28	1.97 to 9.18	0.006	0.37	0.15 to 0.92	0.033

* Age was assessed at start of tamoxifen therapy.
**Missing data not reported

Table S-2. Logistic regression analysis for objective response of PSAT1 stained tumors.

		Univariate			Multivariate		
	N	**OR**	**95% CI**	***P***	**OR**	**95% CI**	***P***
**PSAT1**							
**Negative**	236	1.00					
**Positive**	25	0.26	0.15 to 1.81	0.304			
**Age***							
**≤ 55 years**	99	1.00			1.00		
**> 55 years**	162	2.38	1.18 to 4.79	0.016	2.40	1.15 to 4.99	0.019
**Disease-free survival**							
**≤ 12 months**	41	1.00					
**> 12 months**	220	1.03	0.44 to 2.39	0.943			
**Dominant site of relapse**							
**Loco-regional**	28	1.00			1.00		
**Bone**	104	0.13	0.50 to 0.34	< 0.001	0.13	0.05 to 0.34	< 0.001
**Visceral**	56	0.24	0.91 to 0.66	0.005	0.22	0.08 to 0.61	0.003
**Bone and other**	73	0.26	0.10 to 0.66	0.005	0.25	0.10 to 0.65	0.005
**PgR****							
**Negative**	69	1.00					
**Positive**	191	1.61	0.76 to 3.42	0.214			
**Her2 status****							
**Negative**	210	1.00					
**Positive**	49	0.87	0.39 to 1.94	0.740			
**Tumor differentiation****							
**Good**	42	1.00					
**Moderate**	143	0.45	0.20 to 1.02	0.056			
**Poor**	75	0.73	0.31 to 1.73	0.479			

* Age was assessed at start of tamoxifen therapy.
**Missing data not reported

Table S-3. KEGG pathways associated to PSAT1 expression.

KEGG	comparative	holm	p-value	Statistic	Exp	sd
Glycine, serine and threonine metabolism	0.001	1.11E-06	8.21E-09	4.259	0.649	0.240
Cytokine – cytokine receptor interaction	0.005	1.52E-03	1.13E-05	3.144	0.649	0.235
Olfactory transduction	0.006	2.40E-03	1.81E-05	4.155	0.649	0.340
Jak-STAT signaling.pathway	0.019	4.72E-03	3.57E-05	2.456	0.649	0.207
Glycosphingolipid.biosynthesis..neolactoseries	0.030	2.12E-02	1.60E-05	2.705	0.649	0.314
Leukocyte.transendothelial.migration	0.050	2.17E-02	1.70E-04	2.401	0.649	0.240
Hematopoietic.cell.lineage	0.026	3.88E-02	3.10E-04	2.950	0.649	0.308
Tolllike.receptor.signaling.pathway	0.023	4.02-02	3.22E-04	2.847	0.649	0.299

Acronyms: KEGG: Kyoto encyclopedia of genes and genomes; sd: standard deviation.

Supplementary material – Supplementary tables:

Table S-9. Clinical information of ER positive patients included in the TMA.

	**Patients (%)**
**All patients**	279 (100.0)
**Age***	
**≤ 55 years**	106 (37.9)
**> 55 years**	173 (62.1)
**Menopausal status***	
**Premenopausal**	73 (26.2)
**Postmenopausal**	206 (73.8)
**Tumor size**	
**T1 (≤2cm)**	119 (42.6)
**T2 (2-5cm) + Tx**	137 (49.1)
**T3 (>5cm) + T4**	23 (8.3)
**Tumor differentiation****	
**Good/Moderate**	199 (71.3)
**Poor**	79 (28.3)
**Unknown**	1 (0.4)
**Involved lymph nodes†**	
**0**	96 (34.4)
**≥ 1**	174 (62.4)
**Disease free interval**	
**≤ 12 months**	44 (15.8)
**> 12 months**	235 (84.2)
**Dominant site of relapse**	
**Loco-regional**	29 (10.4)
**Bone**	115 (41.2)
**Visceral**	58 (20.8)
**Bone and other**	77 (27.6)
**PgR†**	
**Negative**	74 (26.5)
**Positive**	203 (72.8)

* Age and menopausal status were assessed at start of tamoxifen therapy.
** Tumor differentiation was evaluated through Scarff-Bloom-Richardson grading system
†Missing data not reported

Acronyms: PgR: progesterone receptor

Table S-10. Clinical Information of patients included in the gene expression cohort.

	Total*
***N* patients**	155 (100.0)
**Age****	
**≤ 55 years**	67 (43.2)
**> 55 years**	88 (56.8)
**Menopausal status****	
**Premenopausal**	49 (31.6)
**Postmenopausal**	106 (68.4)
**Tumor size**	
**T1 (≤ 2cm)**	44 (28.4)
**T2 (2-5cm) + Tx**	92 (59.3)
**T3 (> 5cm) + T4**	19 (12.3)
**Tumor differentiation**	
**Good/Moderate**	44 (28.4)
**Poor**	16 (10.3)
**Unknown**	95 (61.3)
**Involved lymph nodes†**	
**0**	82 (52.9)
**≥ 1**	64 (41.3)
**Disease free interval**	
**≤ 12 months**	33 (21.3)
**> 12 months**	122 (78.7)
**Dominant site of relapse**	
**Loco-regional**	18 (11.6)
**Bone**	85 (54.9)
**Visceral**	27 (17.4)
**Bone and other**	25 (16.1)
**PgR†**	
**Negative**	32 (20.6)
**Positive**	116 (74.8)

* Data are displayed as *N* (percentage).
** Age and menopausal status were assessed at start of tamoxifen therapy.
†Missing data not reported

Supplementary Figure Legends:

Figure S-1. Comparison analysis of PSAT1 protein and mRNA expression.

PSAT1 mRNA levels measured by RT-qPCR (n = 56) and Affymetrix (n = 31) were stratified according to PSAT1 protein levels measured by IHC (i.e. positive vs negative), and differences in mRNA levels were assessed by Mann-Whitney test. A significant difference was observed between PSAT1 mRNA levels measured by RT-qPCR (Mann-Whitney $P = 0.009$; pane A), while no difference was observed between mRNA levels measured by Affymetrix (Mann-Whitney $P = 0.133$; panel B).

Figure S-2. Correlation analysis of PSAT1 mRNA levels measured by Affymetrix and RT-qPCR.

For a panel of 122 tumors, PSAT1 mRNA levels were measured by both RT-qPCR and Affymetrix chip technologies. Spearman correlation analysis showed a moderate-strong correlation between mRNA levels measured with different platforms (Spearman r = 0.742; $P < 0.001$).

Figure S-3. Global test analysis of combined public datasets stratified according to PSAT1 expression.

The ER positive, lymph node negative subset of samples included in the combined publicly derived dataset was analyzed by global test (n = 404; database: KEGG; stratification criterion: PSAT1 median expression). Figure represents the enrichment analysis results of genes belonging to the Cytokine-cytokine receptor interaction (Holm-Bonferroni P = 1.45E-04; Panel A) and the Jak-STAT signaling (Holm-Bonferroni P = 1.75E-07; Panel B) pathways. Bar charts (left) represent enriched genes in each pathway, with red and green columns representing the association to high and low expression of PSAT1, respectively. Heatmaps of most significant genes (enrichment statistic $P < 0.01$; genes are ordered based on decreasing average expression) in relation to PSAT1 expression are displayed on the right.

Figure S-4. Analysis for association of PSAT1 to the TIL gene signature.

Log2 mRNA expression of the 152 genes belonging to the TIL signature was derived out of our gene expression data set (n = 155). PSAT1 levels were correlated to average TIL signature expression in every sample (A),

showing weak positive correlation. A significant enrichment of PSAT1 mRNA level was also found in the high TIL signature group of patients (B).

Figure S-5. Assessment of PSAT1 levels by immunohistochemistry and qPCR on breast cancer cell lines.

Breast cancer cell lines included in the TMA were used as controls. PSAT1 protein (IHC) and mRNA (RT-qPCR) levels were assessed in CAMA, MM175, EVSA-T and DU4475 breast cancer cell lines. Panels A-D display PSAT1 IHC stainings, while panel E displays PSAT1 mRNA levels measured by RT-qPCR.

Supplementary Figures

Supplementary Figure 1.

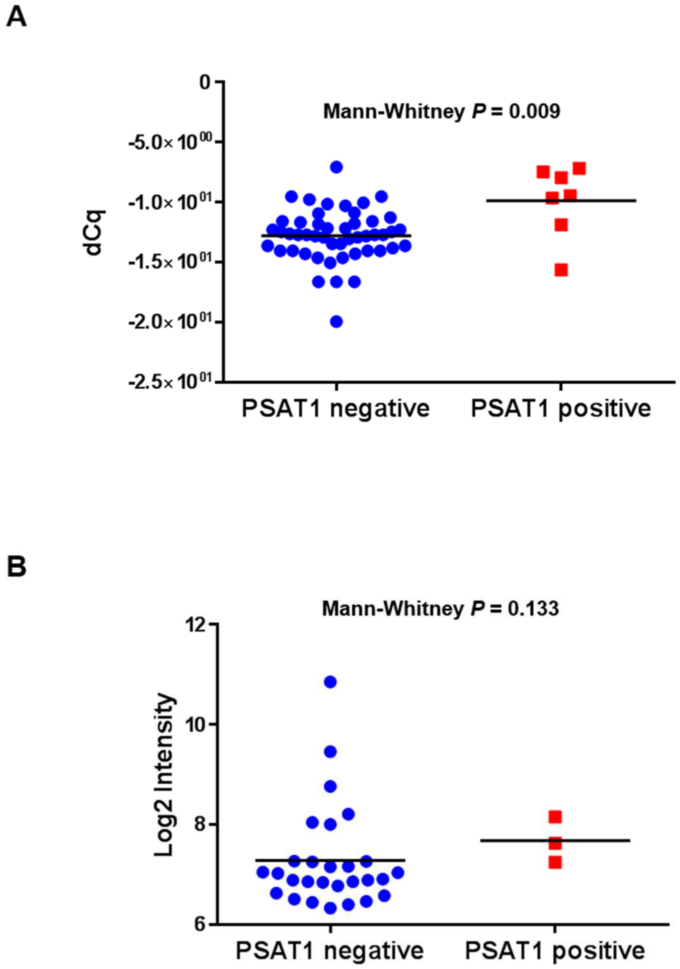

Supplementary Figure 2.

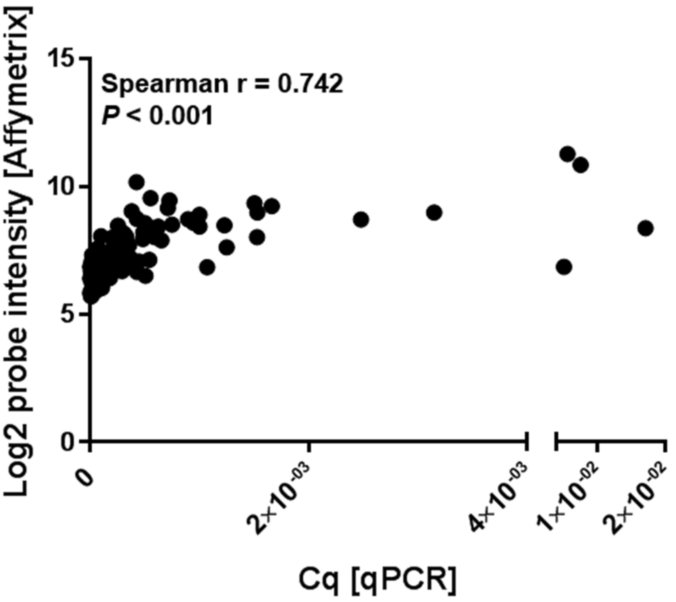

Supplementary Figure 3.

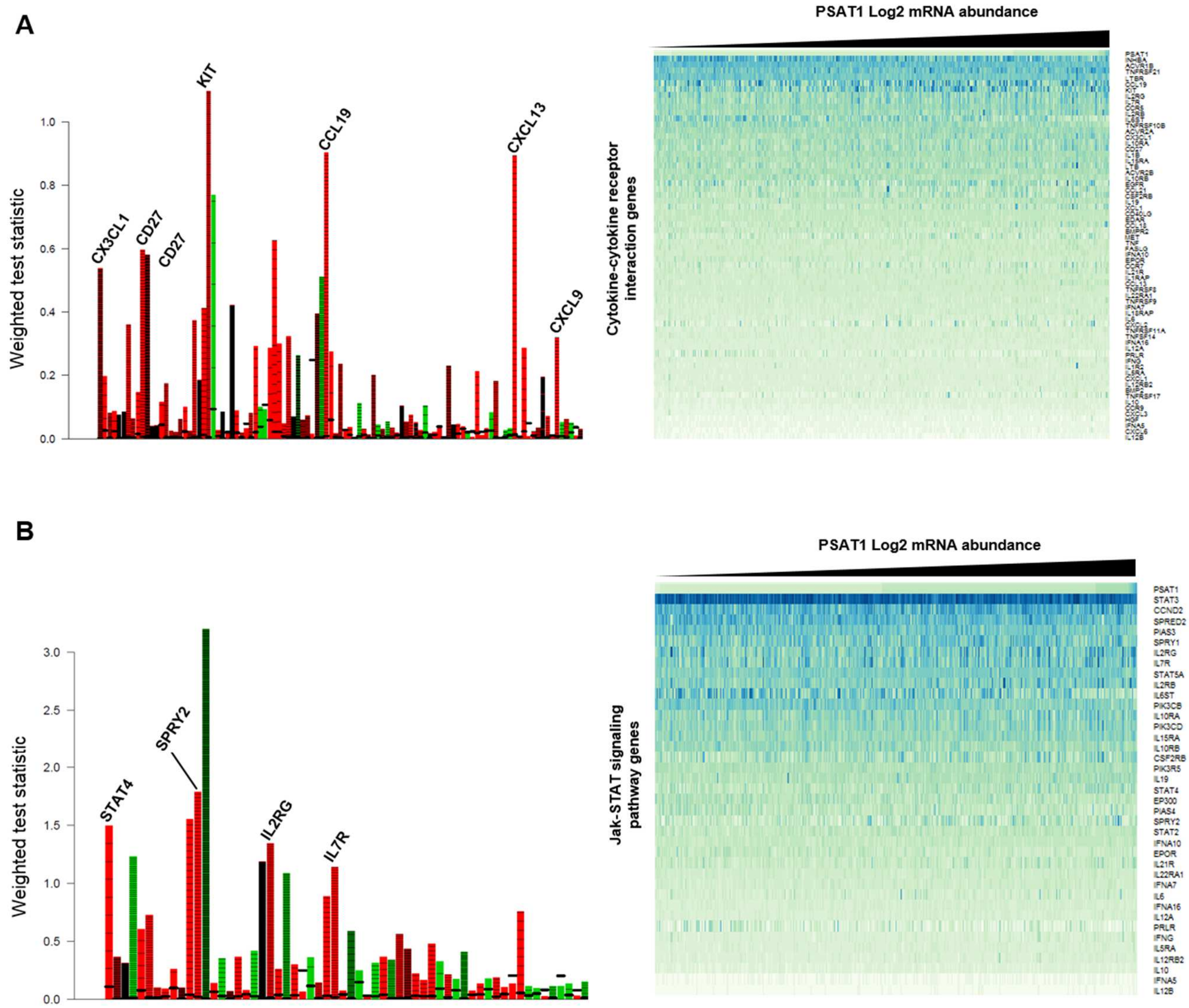

Supplementary Figure 4.

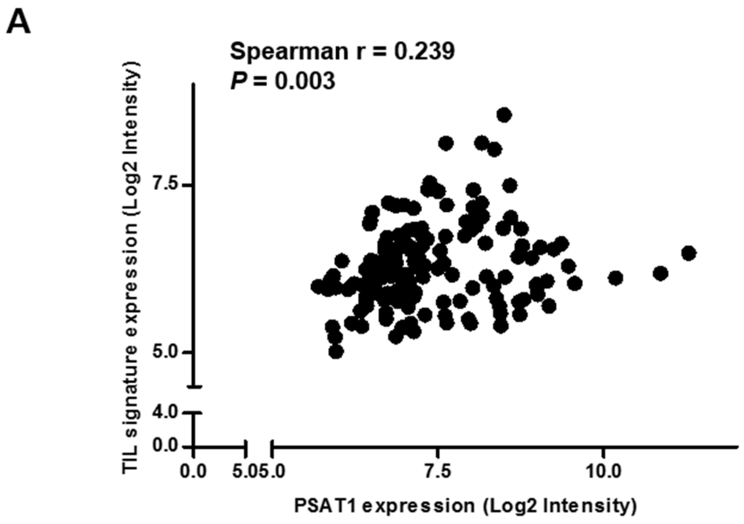

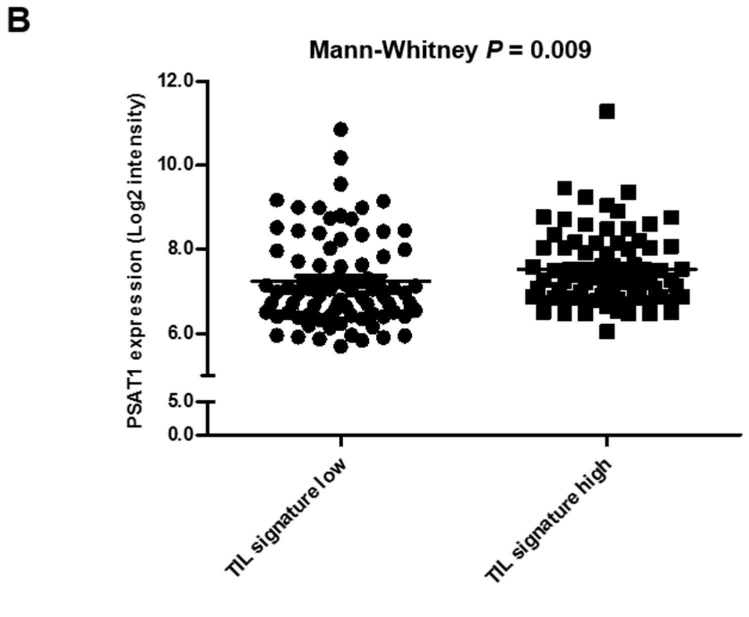

Supplementary Figure 5.

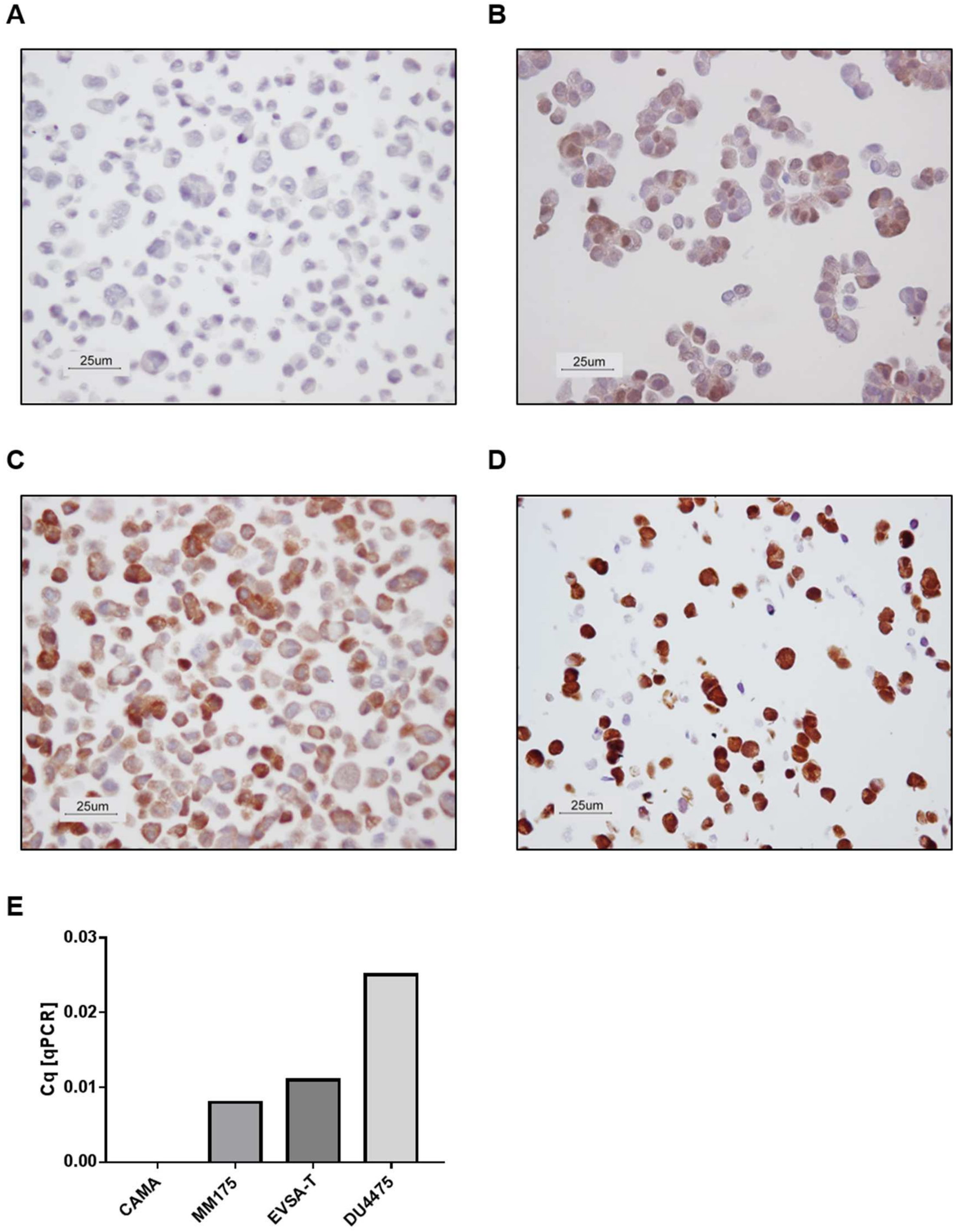

Supplement: Supplementary file 1 — Supplementary material [file 41598_2017_2296_MOESM1_ESM.pdf]
